# A spatially heterogeneous network-based metapopulation software model applied to the simulation of a pulmonary tuberculosis infection

**DOI:** 10.1007/s41109-018-0091-2

**Published:** 2018-08-23

**Authors:** Michael J. Pitcher, Ruth Bowness, Simon Dobson, Stephen H. Gillespie

**Affiliations:** 1 School of Computer Science, University of St Andrews, North Haugh, St Andrews, UK; 2 School of Medicine, University of St Andrews, North Haugh, St Andrews, UK

**Keywords:** Complex networks, Metapopulation, Spatial heterogeneity, Tuberculosis, In-host modelling, Computational biology

## Abstract

Tuberculosis (TB) is an ancient disease that, although curable, still accounts for over 1 million deaths worldwide. Shortening treatment time is an important area of research but is hampered by the lack of models that mimic the full range of human pathology. TB shows distinct localisations during different stages of infection, the reasons for which are poorly understood. Greater understanding of how heterogeneity within the human lung influences disease progression may hold the key to improving treatment efficiency and reducing treatment times.

In this work, we present a novel *in silico* software model which uses a networked metapopulation incorporating both spatial heterogeneity and dissemination possibilities to simulate a TB infection over the whole lung and associated lymphatics. The entire population of bacteria and immune cells is split into a network of patches: members interact within patches and are able to move between them. Patches and edges of the lung network include their own environmental attributes which influence the dynamics of interactions between the members of the subpopulations of the patches and the translocation of members along edges.

In this work, we detail the initial findings of a whole-organ model that incorporates distinct spatial heterogeneity features which are not present in standard differential equation approaches to tuberculosis modelling. We show that the inclusion of heterogeneity within the lung landscape when modelling TB disease progression has significant outcomes on the bacterial load present: a greater differential of oxygen, perfusion and ventilation between the apices and the basal regions of the lungs creates micro-environments at the apex that are more preferential for bacteria, due to increased oxygen availability and reduced immune activity, leading to a greater overall bacterial load present once latency is established.

These findings suggest that further whole-organ modelling incorporating more sophisticated heterogeneities within the environment and complex lung topologies will provide more insight into the environments in which TB bacteria persist and thus help develop new treatments which are factored towards these environmental conditions.

**Electronic supplementary material:**

The online version of this article (10.1007/s41109-018-0091-2) contains supplementary material, which is available to authorized users.

## Introduction

Tuberculosis (TB) is ranked by the World Health Organization as the world’s deadliest infectious disease, accounting for over 1 million deaths in 2016 (World Health Organization 2017). Despite TB being an ancient disease and knowledge of effective treatments for it being available since the 1940s, TB remains prevalent, mostly due to its complex pathology within the human body which is still not fully understood. Treatment for TB infection is a long and arduous process, with the standard regimen for drug-susceptible forms of TB requiring a minimum of 6 months of multiple-drug chemotherapy. This lengthy duration poses a possible global threat as incomplete or inadequate treatment can lead to the development of drug resistance within the *Mycobacterium tuberculosis* (Mtb) bacilli (Gillespie 2002), the causative agent for TB infection. Reducing the length of treatment regimens is thus of great importance in TB, as this could improve adherence and reduce emergence of resistance (Gillespie et al. 2014). Creation of improved regimens requires a greater understanding of the disease pathology, including the interactions that occur between the invading bacilli and the host immune responses as well as the environments that these interactions occur upon.

It is known that TB disease shows differing localisations at different stages, with the initial infection often occurring in the basal regions of the lung. This infection is typically resolved by the immune system containing, but not eradicating, the bacteria, known as latent TB. In cases where this latent TB breaks down, a new disease is established, known as post-primary TB, and this typically occurs in the lung’s apical regions. The cause of these localisations has typically been attributed to the differences in environmental conditions throughout the lung: with the high ventilation of the lower regions resulting in an increased likelihood of particle deposition and thus initial infection there; whilst the environmental factors of the apices provide a favourable environment for Mtb bacteria to proliferate if they can disseminate there. But exactly how these various environmental conditions each contribute to the establishment of a TB infection, and the means of bacterial dissemination across the lung environment, are still poorly understood.

In this work, we first present a brief background of the pathology of TB infection, focusing on the localisation within the lungs during different stages of infection and the factors that may influence it. We investigate the routes of dissemination that allow bacteria to reach the apical regions and the environmental conditions there that make for a suitable region of bacterial proliferation during post-primary TB. We then present previous *in silico* work on TB in the lungs and show how these models have often focused only on TB at the level of a single lesion and have not explored the macro-level dynamics acting at the scale of the whole organ. We then give a background on how metapopulation modelling can be used to simulate scenarios of populations that are localised to distinct spatial regions. For the main body of this work, we present a novel network-based metapopulation model that incorporates the spatial heterogeneity and dissemination opportunities to explore how they affect bacterial growth during a TB infection. We first present MetapopPy, a generalised framework to create metapopulation models and apply asynchronous time dynamics upon them. This framework is then extended to create a TB-specific model that simulates the lung and surrounding lymphatics as a network, with TB pathology and immunological events applied upon it. We use this model to explore the differences in results on models that use a single patch representation of the lung and those that separate the lung into distinct regions, each of which contains environmental attributes that influence bacterial growth and immune response; thereby creating an environment with regions that may provide bacteria with preferential conditions in which to proliferate, provided they can disseminate there.

## Background

### TB pathology

A TB infection is typically initiated by the inhalation of airborne Mtb bacteria which are deposited within the alveolar tissue of the lung. Once there, bacteria encounter the body’s resident immune cells. Two of the main immune cells that engage in host defence against TB are alveolar macrophages (AMs) and dendritic cells (DCs). AMs are phagocytes, cells whose primary function is to locate, ingest and destroy invading particles that may cause damage to the organ tissue. AMs ingest Mtb efficiently, but Mtb have evolved to inhibit the destructive capabilities of the AM (Pieters 2008), and thus many bacteria that are ingested remain alive inside the cell, which provides a niche for the bacteria to replicate uninhibited (Collins and Kaufmann 2001). These AMs which are ‘infected’ with bacteria have reduced destructive capabilities. Bacteria then proceed to replicate both intracellularly and extracellularly and cause tissue damage; this is known as ‘primary’ TB.

In order to overcome this subversion of the innate immune system, the body must increase the scale and strength of its immune response. This is done through increasing the number of immune cells recruited to the site of infection (Wager and Wormley 2014) and the triggering of an adaptive immune response, a function which is performed by the DCs. These cells come into contact with bacteria and absorb antigens. Doing so cause the DCs to change from an immature state to a mature state, and these mature cells then migrate from the lungs to the lymphatic system (Alvarez et al. 2008; Bousso 2008; Mihret 2012). There, they act as antigen-presenting cells (APCs), whose role is to present antigens to naïve T-cells and thus trigger an adaptive immune response. This presentation causes naïve T-cells to differentiate and activate, and then proceed into the lung tissue through the blood in order to help fight the infection. Whilst DCs are viewed as the primary APC in TB infection, it has been shown that AMs may also function as antigen presenters (Kirby et al. 2009).

Within the lungs, the presence of T-cells causes a profound impact on the immune response to TB through a variety of functions. These cells release cytokines - signalling chemicals which cause the AMs that come into contact with them to change their behaviour. Uninfected macrophages may be triggered by these cytokines to activate; doing so changes the macrophage state to an activated one whereby the macrophage has greatly enhanced bactericidal capabilities (Pieters 2008). T-cells also perform cytotoxic killing of cells that have become infected with bacteria.

The increased immune response results in a battle between host and bacteria that typically (in 95% of cases) results in a stalemate: the immune response can contain bacterial growth but is unable to completely eradicate the bacteria from the body. This state is known as Latent TB, and containment occurs through structures known as granulomas, which are complex arrangements of activated immune cells and fibrosis surrounding infected immune cells and bacteria in the centre, preventing bacterial replication by restricting the space and nutrient supply available. Mtb are able to persist within this granuloma structure by entering a dormant state (Ehlers 2009), whereby the bacteria increase their resistance to chemotherapeutic destruction (Lipworth et al. 2016), but at the cost of also significantly lowering their replication rate. The exact triggers for this process are still unclear. Latent TB patients are asymptomatic and cannot pass infection onto others, and they may remain in this state for years. But latent TB poses a risk of future infection (Gomez and McKinney 2004) in cases where the immune system weakens: the granuloma structures may break down, releasing bacteria and allowing them to replicate and cause tissue damage, known as ‘post-primary’, or ‘reactivation’ TB.

### TB localisation within the lung

TB has been shown to exhibit distinct localisations within the lungs during these different stages of infection (Elkington and Friedland 2015). Upon initial infection, Mtb are typically deposited towards the basal regions of the lungs, due to a difference in ventilation within the lungs caused by the effects of gravity and bronchial tree anatomy (Drake et al. 2015) that forces more inhaled air (and thus also any inhaled particles) into the lower regions (West 2005). However, reactivation TB occurs within the apical regions of the lungs (Murray 2003; Balasubramanian et al. 1994). These findings suggest two important factors concerning pulmonary TB infections: i) the bacteria are able to disseminate within the lung and surrounding systems during infection and ii) the apical regions present an environment that is favourable for bacterial proliferation during post-primary infection. The exact causes for this difference in localisation are poorly understood, both in terms of how the bacteria disseminate and the factors that make the apical region favourable to Mtb.

### Dissemination

Bacterial dissemination may occur through a variety of possible routes. When APCs migrate to the lymphatic system to trigger the adaptive immune response, they also provide any internalised bacteria with a means of transport to the lymphatics (Humphreys et al. 2006). From here, it has been hypothesised that the bacteria can either re-enter the lung through damage to the lymphatic tissue (Behr and Waters 2014), or access from the lymphatics to the blood stream may provide an alternative route of dissemination to previously uninfected areas (Hunter 2016), which may be more beneficial to bacterial growth.

### Spatial heterogeneity

The lung environment is heterogeneous, with a variety of environmental attributes differing over regions of the lung. Both ventilation (the amount of air passed into a region of the lung during inhalation) and perfusion (the amount of blood supplied to a region of the lung for oxygen exchange) show regional variances, with both increasing in value towards the lower regions. As perfusion shows a more rapid increase, with much lower perfusion occurring at the apices, this creates a differential in oxygen tension (calculated as ventilation divided by perfusion), with the apical regions being oxygen-rich (as little oxygen exchange occurs) and the basal regions being oxygen-poor (as all oxygen is exchanged).

It has been hypothesised that this localisation of TB to the apical regions is due to these environmental attribute differentials (Goodwin and Des Prez 1983; Murray 2003). Mtb are an aerobic bacilli, requiring oxygen for their metabolism, and a hypoxic environment has been shown to be a trigger to convert the bacilli into a dormant state (Hammond et al. 2015; Lipworth et al. 2016). Thus, the relatively higher partial pressure of oxygen at the apical regions may mean the bacteria present there are able to enter a replicating state easier and thus proliferate faster. Perfusion differentials may also impact disease progression, as reduced perfusion results in a weakened immune response due to the reduced recruitment of immune cells through the blood (Hunter et al. 2014).

Furthermore, transfer of immune cells to the lymphatics also serves as a means of bacteria clearance from the lungs. The rate of clearance shows heterogeneity within the lungs, with the apices having a reduced clearance rate (Goodwin and Des Prez 1983). This may result in fewer bacteria being trafficked out of the lungs and thus increasing the bacterial load at the apices.

### *In silico* modelling

These factors of dissemination and heterogeneity are hypothesised to affect the progression and localisation of a TB infection, but their exact contributions are still controversial (Elkington and Friedland 2015), and studies into how these factors impact TB disease are difficult due to the lack of fully viable models: in vivo animal models often do not display completely human-like pathology, whilst in vitro models cannot replicate the broad spectrum of physiological conditions found within the human body (Guirado and Schlesinger 2013). *In silico* mathematical and computational models provide the means to simulate environmental conditions that cannot be produced with real-world studies.

With regard to TB, *in silico* modelling has been used to model TB disease within-host (see Kirschner et al. (2017) for an extensive review), particularly to investigate factors surrounding the development of a single lesion in the lung (Guirado and Schlesinger 2013), including the important factors in granuloma formation and control (Segovia-Juarez et al. 2004; Cilfone et al. 2013) as well as models that simulate the transfer of immune cells between the lung and the lymphatics during infection (Marino and Kirschner 2004; Marino et al. 2010). Previous work has also investigated the effects of bacterial cell state on the formation of a lesion (Bowness et al. 2018). The majority of TB in-host models only simulate a small region of lung tissue (enough to contain a single lesion) or treat the lung as a homogeneous environment with identical environmental conditions throughout the simulated landscape; to our knowledge, there are no models that attempt to determine how the heterogeneities across the whole human lung influence the progression of a TB infection or include dissemination of bacteria between these different regions of the lung.

### Metapopulations

Spatial heterogeneities in the landscape have been shown to have profound effects on the dynamics of populations within an environment. Real-world populations in a variety of scenarios are not well-mixed; populations tend to cluster into distinct spatial regions (‘patches’), thus creating a ‘population of populations’ or ‘metapopulation’. In typical metapopulation modelling, members within the subpopulation present at a patch are assumed to be well-mixed. Each patch is spatially distinct, but patches may influence each other through the dispersal of members between patches. Thus, this combination of patches and dispersal routes can be seen as a network, where patches represent the nodes of the network and dispersal routes represent the edges. Understanding how the environmental factors that differ between patches affect the interactions that take place upon patches and between them can provide greater insight into the overall system dynamics, and can be utilised to create effective control measures in the case of disease containment (Levins 1969).

Metapopulations have seen many implementations in a variety of different disciplines, with significant use in the fields of ecology and epidemiology. These models have explored the concepts of colonisation and extinction of a species (Hanski and Gilpin 1991; Grenfell and Harwood 1997), where each patch is either occupied by the species or is unoccupied, with an occupied patch providing the opportunity for its immediate, unoccupied neighbours to become occupied as well (colonisation) or for the patch to become unoccupied (extinction). Local extinction of a species across all patches results in a global extinction of the species. Epidemiological models of this nature take a similar approach, with occupation of a patch indicating the presence of a disease within the host members located there (Foley et al. 1999). Rather than an occupied/unoccupied view, some metapopulation models have used a more granular approach, whereby various subpopulations of species are modelled explicitly within the patches and allowed to interact with one another. These are particularly used in epidemiology (Arino and van den Driessche 2006; Hickson et al. 2012), where the species represent individuals in different states of disease or susceptibility and have been used to show that greater understanding of the differentials within the global population can allow researchers to understand and better control the dynamics of global infections (Helbing et al. 2014) and how spatial heterogeneities in aspects like contact rates, vaccination uptake and demographics can alter the effectiveness of intervention programs to prevent disease transmission (Wang et al. 2016).

Ganguli et al. (2005) use this approach in modelling the formation of a single TB lesion, with a grid of compartments modelling a small portion of alveolar tissue, each of which contains a variety of species including bacteria and various immune cells. Their metapopulation model incorporates spatial heterogeneity by restricting the interactions between agents of the system to only those in the same spatial compartment, with some agents able to move between compartments. Environmental heterogeneity is included through the diffusion of chemokine (a signalling chemical produced by immune cells) from the source of infection to adjacent compartments. However, their model simulates the formation of a single lesion and does not incorporate environmental factors such as oxygen and perfusion differentials as discussed previously.

## Networked metapopulation model framework

Metapopulation modelling provides the means of modelling scenarios where the main population is localised to specific distinct spatial regions, which may differ from one another in terms of environmental resources. TB infections within the lung can be seen as such a scenario, with bacteria and immune cells being restricted to different regions of the lung, each of which varies in terms of attributes such as oxygen availability and blood perfusion. Therefore, to model the whole-organ impact of a TB infection within the lung, a metapopulation model is appropriate, as it allows the incorporation of spatial heterogeneity within the environment on which a population resides. In this section, we detail the creation of a generalised framework for creating metapopulation models called *MetapopPy*, which is then used as the basis of a specific model to simulate TB infections within the human lungs. *MetapopPy* is written in Python and allows for the simple creation of networked metapopulational simulation models. As this is an abstract framework, the models created can be applied to any field of study involving spatially distributed populations. This modelling framework is described in full detail in the Additional file 1.

The framework consists of a metapopulation network of patches, connected by edges, and a set of events. Each patch of the network contains a subset of the entire system population divided into compartments: members of the subpopulation may belong to only one compartment at any time, but may transfer between compartments based on the system dynamics. The events are attached to patches based on where they may occur. The event modelling within the MetapopPy system uses the Gillespie Algorithm^1^ to perform events asynchronously (Gillespie 1976). Although this algorithm was initially developed to model chemical reactions, it is applicable to any scenario that involves interactions between members of a population, including infection spreading over networks (Vestergaard and Génois 2015).

When a simulation begins, each event is initialised with a static reaction parameter, which can be interpreted as the rate of a single reaction of the given event in the given unit time, and each event contains a function to calculate a state variable, which can be interpreted as the number of possible reactions of the event at the current time. The state variable function is applied to every patch in the network, and the sum of the results of these is the event’s state variable.

From these, a rate for every event is calculated, by taking the product of the event’s reaction parameter and its current state variable. Having calculated the rate for every event of the model, an event is chosen probabilistically to be performed (with a higher rate resulting in a greater chance of selection). A timestep, *τ*, for this event to occur is then chosen using Eq. 1 (as per Gillespie (1976)), with *a* being the total sum of the rate of all events, and *r* being a randomly generated number in the range (0,1).

1 $$  \tau = \frac{1}{a} ln \left(\frac{1}{r}\right)  $$

The chosen event is then performed, and updates the network in its defined manner. The simulation time is then incremented by *τ* and the process repeats, until a set time-limit is exceeded or there is no possibility of any event occurring (i.e. *a*=0).

## Pulmonary TB infection model

The above MetapopPy framework has been extended to create TBMetapopPy, a metapopulation-based model that simulates a tuberculosis infection within the human pulmonary system, investigating how spatial heterogeneities of environmental factors within the lungs and dissemination of bacteria within the lung and between the lung and the lymphatics influence the spread of an infection. This model follows the same three-package structure as above, with a specific environment and set of events to simulate the early stages of a pulmonary TB infection.

### Network

**Fig. 1:**
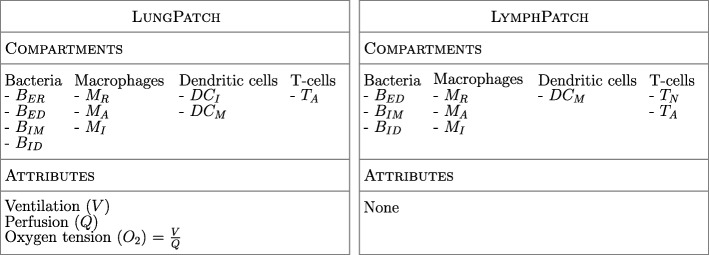
Diagram of the two types of patches used in *TBMetapopPy*, along with the compartments (see Table 1) and the environmental attributes present in each. LungPatch instances contain spatial attributes to allow for the creation of a heterogeneous environment within the lung. Oxygen tension in each patch is always calculated as the division of ventilation by perfusion

*TBMetapopPy* models the environment of the lung and lymphatics - the compartments used represent the total population of bacteria and immune cells, with each compartment denoting a unique cell type and cell state as detailed in Table 1. We define two distinct types of patches: LungPatch, which represents a portion of alveolar tissue found within the lung, and LymphPatch, which simulates the tissue within the lymphatic system. LungPatch instances contain 3 spatial attributes: Ventilation (*V*), Perfusion (*Q*) and Oxygen Tension (*O*_2_), while LymphPatch instances have none. Figure 1 shows the setup of the two types of patches.

**Table 1:** Subpopulation compartments used in *TBMetapopPy*

Cell type	Name	Symbol	Description
Bacteria	Bacterium extracellular replicating	*B*_*ER*_	Bacteria present on the alveolar tissue which have a high replication metabolism
	Bacterium extracellular dormant	*B*_*ED*_	Bacteria present on the alveolar tissue which have a low replication metabolism
	Bacterium intracellular macrophage	*B*_*IM*_	Bacteria which are present inside infected macrophages (*M*_*I*_)
	Bacterium intracellular macrophage	*B*_*ID*_	Bacteria which are present inside mature dendritic cells (*DC*_*M*_)
Macrophages	Macrophage resting	*M*_*R*_	Macrophages in a natural state
	Macrophage activated	*M*_*A*_	Macrophages which have been activated by the immune system to increase cytotoxic capabilities
	Macrophage infected	*M*_*I*_	Macrophages which have internalised, and become infected by, bacteria
Dendritic cells	Dendritic cell immature	*D* *C*_*I*_	Dendritic cells in a natural, immature state
	Dendritic cell mature	*D* *C*_*M*_	Dendritic cells which have come into contact with bacteria and have matured into an antigen-presenting state
T-cells	T-cell naïve	*T*_*N*_	T-cells in a natural, unactivated state
	T-cell activated	*T*_*A*_	T-cells which have been activated by antigen presentation

**Fig. 2:**
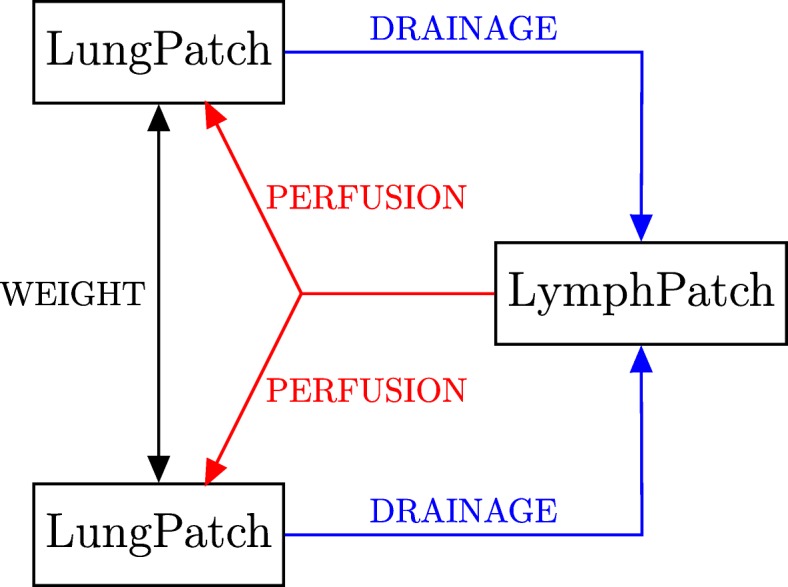
Diagram of the types of edges used in *TBMetapopPy* network, along with the attributes of each. LungEdges (in black) indicate movement of bacteria within the air of the lungs, and thus connect two LungPatch instances together, with a WEIGHT value indicating probability of movement. LymphaticEdges (in blue) indicate the migration of cells from the lung to the lymphatic system, and connect a LungPatch to a LymphPatch. DRAINAGE indicates the flow rate of cells to the lymphatic system. BloodEdges (in red) indicate the movement of bacteria and cells into the lung via the bloodstream and thus connect a LymphPatch to a LungPatch. These edges contain a PERFUSION value, set equal to the perfusion value of the LungPatch

In order to create a network, edges are defined connecting the LungPatch instances together, and joining those instances with the LymphPatch instances, as shown in Fig. 2. Edges also contain environmental attributes influencing the rate of translocation of members between the patches. LungEdge instances contain a WEIGHT attribute to indicate the rate of air flow, and thus the rate of cell movement upon the air, between two LungPatch instances. LymphEdge instances contain a DRAINAGE value to indicate the rate of migration of cells from the lung to the lymphatic system. BloodEdge instances represent the movement of cells from the lymphatics to the blood stream from which they are re-seeded back into the lung. These contain a PERFUSION attribute, set equal to the value of perfusion in the destination LungPatch. This allows for differential lymphocyte (cells originating from the lymphatics) immune responses throughout the lung.

### Events

*TBMetapopPy* contains a number of events to simulate the growth of bacteria and the interactions between the immune cells of the body and the bacteria. These events, along with their respective state variable functions, are described in more detail within the Additional file 1.

In order to investigate the effects of environmental spatial heterogeneity upon TB infection, some of these events are influenced by the environmental attributes present at each patch. For example, we include events to switch bacteria from a replicating state (*B*_*ER*_) to a dormant one (*B*_*ED*_), and conversely from dormancy to actively replicating. These events have state variable functions that are dependent on the oxygen tension level of the patch, which we hypothesise is the main driver between these metabolic states. The switch to dormancy increases with lower oxygen, whilst the switch to a replication state increases with greater oxygen (see Additional file 1: Table S1 State Variable functions). Likewise, events which involve the recruitment of immune cells into lung compartments are scaled by the perfusion value of the patch, and thus a greater immune response can be established in patches where more blood is perfused.

### Dynamics

A TBModel class combines the topology and event packages. The chosen lung topology is initialised and seeded with values for resident immune cells (*M*_*R*_ and *DC*_*I*_ in the LungPatches, *M*_*R*_ and *T*_*N*_ in the LymphPatches) based on the recruitment and death values to calculate average equilibrium levels of these cells. A population of bacteria are seeded, either in a defined location or probabilistically based on *V* values. The simulation proceeds as per the MetapopPy framework until a simulated time limit is reached.

## Results

### Methods

**Fig. 3:**
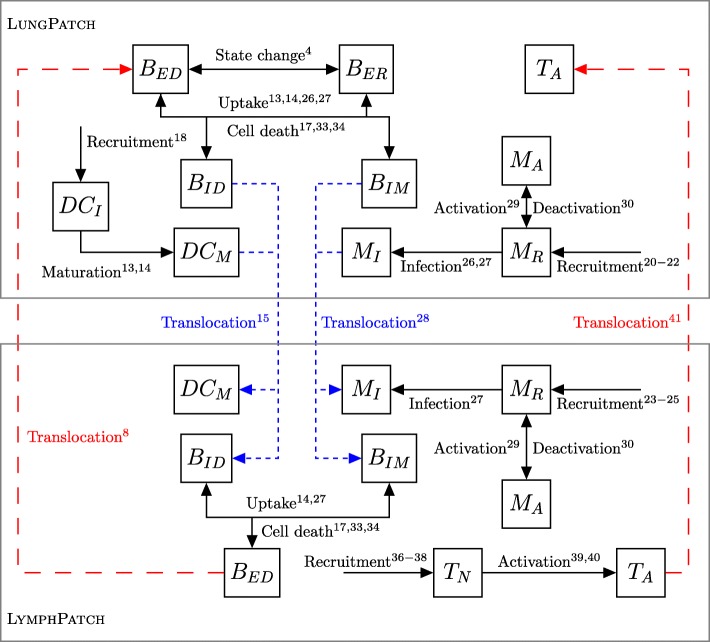
Schematic for the dynamics of TBMetapopPy using topology A. Arrows indicate possible transition routes of members between compartments. Long-dash red arrows indicate translocation across a BloodEdge from lymphatics to the lung, short-dashed blue arrows indicate translocation across a LymphEdge, from the lung to the lymphatics. Numbers for each transition relate to the events that define the transition, as described in the Additional file 1

In order to demonstrate the effects of spatial heterogeneity upon TB infection within the lungs, we performed our experiments against two distinct topologies of the TBMetapopPy model. In the first, topology A, the lung is modelled as a whole homogeneous unit, consisting of just one LungPatch coupled to a LymphPatch which models the lymphatics. A LymphEdge and a BloodEdge allow movement of cells between the two. This topology and the dynamics of the transition of members between compartments is shown in Fig. 3.

**Fig. 4:**
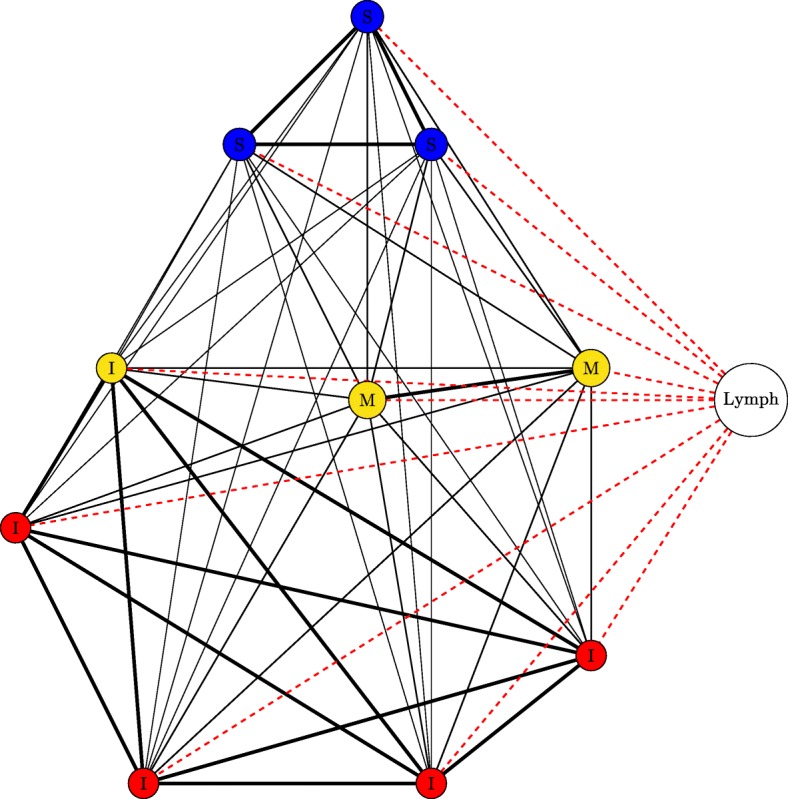
Structure of topology B. The lung is divided into 10 patches. Patches are assigned into zones based on their vertical position (blue = Zone 1, yellow = Zone 2, red = Zone 3). All patches are connected, and patches within the same lobe (S = Superior lobe, M = Middle lobe, I = Inferior lobe) have greater edge width. All lung patches are connected to/from the LymphPatch by a LymphEdge and a BloodEdge (both edges denoted by a single dotted red line)

In the second, topology B, the lung is broken up into distinct instances of the LungPatch class. Each of these patches represents a bronchopulmonary segment - an anatomical division of the bronchial tree which is supplied by a segmental bronchus (Drake et al. 2015). For the purposes of this simulation, we choose to model the right lung, and thus our lung model contains 10 patches (as the right lung contains 10 bronchopulmonary segments). Each of these patches is grouped into one of three zones depending on its approximate vertical position in the lung: patches towards the apex of the lung are placed into the Apical category, patches in the middle regions are placed into the Middle category, whilst patches towards the base are placed into the Basal category. These categories determine the values of the attributes within, as per Table 2. The structure of the bronchi within the lung is that of a tree, with each patch of the model representing all the leaf nodes within a particular branch of the tree. As air in one section should be able to access all other sections (i.e. there is a path between all leaf nodes), we have chosen a fully-connected topology with varying edge weights between different nodes of the network: each bronchopulmonary segment lies within a distinct lobe (of which there are 3 in the right lung), and for this model we assume that movement of bacteria along the air is more likely to occur between patches within the same lobe and less likely between patches in different lobes. Topology B is shown in Fig. 4.

**Table 2:** Values for attributes of West Zones used in simulations of multi-patch topology model, unless specified in the text

	*V*	*Q*	*O*_2_
Apical	0.6	0.5	1.2
Middle	0.8	0.8	1.0
Basal	1.2	1.3	0.923

*V* = Ventilation, *Q* = Perfusion, *O*_2_ = Oxygen tension

As the model is stochastic in nature, all experiments are run over 30 repetitions, with the mean counts of members (and standard deviations) of each compartment over these repetitions calculated at each timestep. For topology B, 5 *B*_*ER*_ members are placed into one of the basal regions at the start of simulation. In topology A models, they are placed in the single lung patch.

We are interested in overall bacterial growth, and our marker for this is the combined total of the *B*_*ER*_, *B*_*ED*_, *B*_*IM*_ and *B*_*ID*_ compartments in each patch of the lung.

### Topology A: homogeneous systems require significant reduction in immune activity in order to establish infection

**Fig. 5:**
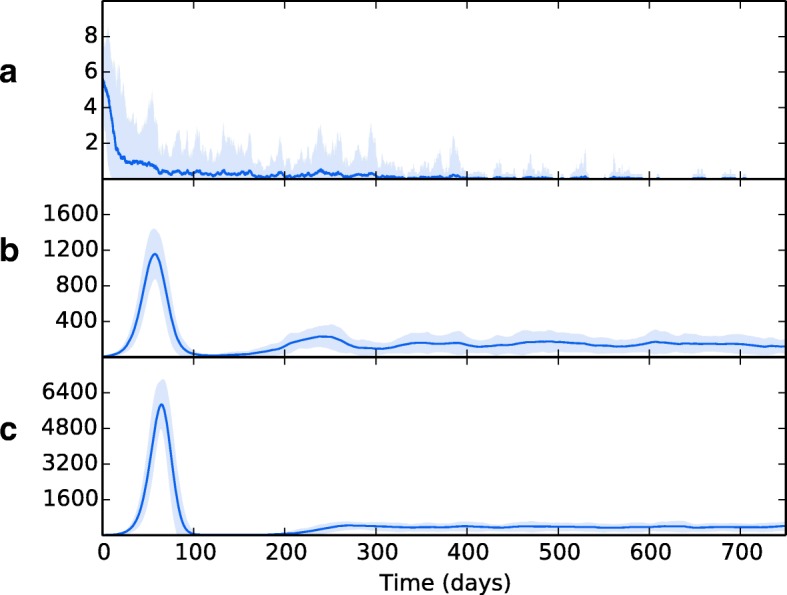
The overall bacterial growth (*B*_*ER*_, *B*_*ED*_, *B*_*IM*_ and *B*_*ID*_) within a single patch topology (Topology A). Line is mean, shading is standard deviation. **a** shows results using event parameters as described in Tables 3, 4, 5 and 6. Infection is contained quickly and virtually eradicated. **b** shows the effects of a minimal reduction in cell recruitment (*R*_*D*_ = 50, $R_{M\_lung}$ = 40) and **c** shows the effects of a major reduction in cell recruitment (*R*_*D*_ = 25, $R_{M\_lung}$ = 20). As recruitment is reduced, the spike of initial infection (at around 50 days) increases. In both cases, the infection is contained and remains at a reasonably steady state thereafter (latency)

Figure 5a shows the overall bacterial growth over time for the model using topology A and parameters as per Tables 3, 4, 5 and 6. For this model, we set the *V* and *Q* values of the patch to 9.0 and 9.1 respectively, chosen in order to match the sum of the total ventilation and perfusion over an entire lung as seen in topology B. Bacterial growth is severely limited, as the bacteria population is overwhelmed by the numbers of immune cells, and the entire population of bacteria is eradicated within 750 days.

**Table 3:** Bacterial event parameters used for experiments, unless specified in the text

Parameter	Description	Value (per day)	Source
*M*_*Cap*_	Maximum number of bacteria that can reside in a macrophage	50	(Marino and Kirschner 2004)
*θ*	Intracellular bacteria replication sigmoid	2	(Marino and Kirschner 2004)
*R*_*ER*_	Replication rate of *B*_*ER*_	0.25	(Wigginton and Kirschner 2001)
*R*_*ED*_	Replication rate of *B*_*ED*_	0.005	(Wigginton and Kirschner 2001)
*R*_*IM*_	Replication rate of *B*_*IM*_	0.005	Estimated
$D_{B\_R}$	Destruction rate of *B*_*ER*_ and *B*_*ED*_ by *M*_*R*_	1.25E-8	(Marino and Kirschner 2004)
$D_{B\_A}$	Destruction rate of *B*_*ER*_ and *B*_*ED*_ by *M*_*A*_	1.25E-4	Estimated
*C*	Rate of conversion between *B*_*ED*_ and *B*_*ER*_	1	Estimated
*H*_*C*_	Half-sat value of *O*_2_ for conversion between *B*_*ED*_ and *B*_*ER*_	1	Estimated
*λ*	Sigmoid value for conversion between *B*_*ED*_ and *B*_*ER*_	1	Estimated
$T_{B\_Lung}$	Rate of translocation of *B*_*ER*_ and *B*_*ED*_ along LungEdge	0.001	Estimated
	(within lung)		
$T_{B\_Blood}$	Rate of translocation of *B*_*ER*_ and *B*_*ED*_ along BloodEdge	0.05	Estimated
	(lymph to lung)

**Table 4:** Dendritic cell event parameters used for experiments, unless specified in the text

Parameter	Description	Value (per day)	Source
*D*_*DCI*_	Rate of death of *DC*_*I*_	0.01	(Marino and Kirschner 2004)
*D*_*DCM*_	Rate of death of *DC*_*M*_	0.02	(Marino and Kirschner 2004)
*U*	Rate of uptake of *B*_*ER*_ and *B*_*ED*_ by *DC*_*I*_, causing maturation	1E-7	(Marino and Kirschner 2004)
*R*_*D*_	Standard recruitment rate of *DC*_*I*_ into lung	500	(Marino and Kirschner 2004)
*E*_*D*_	Enhanced recruitment rate of *DC*_*I*_ into lung caused by *B*_*ED*_	0.02	(Marino and Kirschner 2004)
	and *B*_*ER*_		
*H*_*D*_	Half-sat for enhanced recruitment rate of *DC*_*I*_ into lung caused by	6.5E5	(Marino and Kirschner 2004)
	*B*_*ED*_ and *B*_*ER*_		
*T*_*D*_	Rate of translocation of *DC*_*M*_ along LymphEdge (lung to lymph)	0.5	(Marino and Kirschner 2004)

**Table 5:** Macrophage event parameters used for experiments, unless specified in the text

Parameter	Description	Value (per day)	Source
*A*_*M*_	Rate of activation/deactivation of *M*_*R*_ by *T*_*A*_	0.3	(Marino and Kirschner 2004)
*H*_*MA*_	Half-sat value for activation of *M*_*R*_ by *T*_*A*_	5E2	Estimated
*I*	Rate of macrophage infection by *B*_*ER*_ and *B*_*ED*_	0.4	(Marino and Kirschner 2004)
*H*_*MI*_	Half-sat value for macrophage infection	1E6	(Marino and Kirschner 2004)
*D*_*MR*_	Death rate of *M*_*R*_	0.01	(Marino and Kirschner 2004)
*D*_*MA*_	Death rate of *M*_*A*_	0.01	(Marino and Kirschner 2004)
*D*_*MI*_	Standard death rate of *M*_*I*_	0.01	(Marino and Kirschner 2004)
*B*	Rate of bursting of *M*_*I*_ due to *B*_*IM*_	0.25	(Marino and Kirschner 2004)
$R_{M\_lung}$	Standard rate of recruitment of *M*_*R*_ into lung	400	(Wigginton and Kirschner 2001)
$R_{M\_lymph}$	Standard rate of recruitment of *M*_*R*_ into lymph	53.465	(Marino et al. 2010)
$E_{M\_MI}$	Enhanced rate of recruitment of *M*_*R*_ into lung by *M*_*I*_	0.0056	(Marino and Kirschner 2004)
$E_{M\_MA}$	Enhanced rate of recruitment of *M*_*R*_ into lung by *M*_*A*_	0.04	(Marino and Kirschner 2004)
*T*_*M*_	Rate of translocation of *M*_*I*_ along LymphEdge (lung to lymph)	0.25	Estimated

**Table 6:** T-cell event parameters used for experiments, unless specified in the text

Parameter	Description	Value (per day)	Source
*K*	Rate of destruction of *M*_*I*_ by *T*_*A*_	1.3	(Marino and Kirschner 2004)
*H*_*K*_	Half-sat value for destruction of *M*_*I*_ by *T*_*A*_	0.5	(Marino and Kirschner 2004)
$A_{T\_MI}$	Rate of T-cell activation by antigen presentation by *M*_*I*_	1E-5	Estimated
$A_{T\_DCM}$	Rate of T-cell activation by antigen presentation by *DC*_*M*_	1E-5	(Marino and Kirschner 2004)
*D*_*TN*_	Rate of death of *T*_*N*_	0.102	(Marino and Kirschner 2004)
*D*_*TA*_	Rate of death of *T*_*A*_	0.3333	(Marino and Kirschner 2004)
*R*_*T*_	Standard rate of recruitment of *T*_*N*_ into lymph	1000	(Marino and Kirschner 2004)
$E_{T\_DCM}$	Enhanced rate of recruitment of *T*_*N*_ into lymph due to *DC*_*M*_	0.1	(Marino and Kirschner 2004)
$E_{T\_MI}$	Enhanced rate of recruitment of *T*_*N*_ into lymph due to *M*_*I*_	0.1	Estimated
*T*_*T*_	Rate of translocation of *T*_*A*_ along BloodEdge (lymph to lung)	0.6	(Marino and Kirschner 2004)

We then investigated the conditions necessary to establish a bacterial presence. In order to do this, we reduced the recruitment rates of dendritic cells and macrophages (*R*_*Dendritic*_ and $R_{M\_lung}$ respectively) by arbitrary factors of 10 (shown in Fig. 5b) and then 20 (shown in Fig. 5c). This severe weakening of the immune system recruitment throughout the course of infection is enough to allow TB to establish a presence within the lungs.

**Fig. 6:**
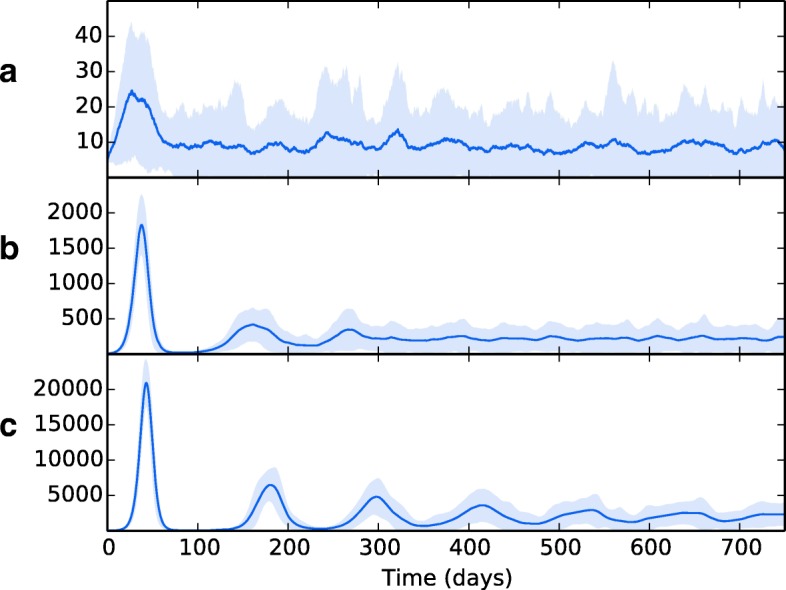
The overall bacterial growth (*B*_*ER*_, *B*_*ED*_, *B*_*IM*_ and *B*_*ID*_) within a single patch topology (Topology A) when perfusion is reduced to **a** 4.5, **b** 1.3, **c** 0.5. Line is mean, shading is standard deviation. Reducing perfusion allows a bacterial presence to be established. In **a** no major initial infection takes place and the infection level remains fairly constant throughout. In **b** a pattern similar to the reduced recruitment rate in Fig. 5 is seen. In **c**, the reduced perfusion results in a saw-tooth pattern, whereby latency is now involves an oscillating, rather than constant, level of bacteria

This was explored further by instead reducing the *Q* value of the lung patch in topology A. This has the effect of also weakening the adaptive immune response, which requires perfusion in order to increase macrophage and dendritic cell numbers and allow t-cells to access the lung. Figure 6a and b show the effects of reducing the *Q* value of the single patch to 4.5 and 1.3 respectively. Reducing perfusion allows the establishment of a bacterial presence, with a higher bacterial load being established with perfusion reduced to very low levels. In Fig. 6c, perfusion of the whole lung is reduced to levels used in the apical regions of topology B (i.e. 0.5). Here, a bacterial presence is firmly established. This suggests that lack of perfusion may be the main cause for bacterial growth as low-perfused patches experience high bacterial growth in both topologies.

### Topology B: spatial heterogeneity increases overall bacterial load

**Fig. 7:**
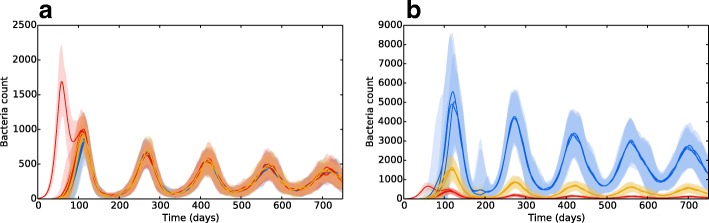
The overall bacterial growth (*B*_*ER*_, *B*_*ED*_, *B*_*IM*_ and *B*_*ID*_) upon a multiple patch topology. Patches are grouped by spatial location (base, middle and apex). Line is mean, shading is standard deviation. All simulations use event parameters as per Tables 3, 4, 5 and 6. **a** shows the results of a network that includes no spatial heterogeneity - initial infection spikes in the initial, basal patch and spreads to other patches, where infection is established at consistent levels throughout. **b** shows the effects of introducing heterogeneity (with values as per Table 2). Infection spreads to other patches, with the bacterial load dependent on the spatial location

In order to explore the effects of spatial heterogeneity (both in the separation of the overall lung population into discrete patches and the variation of resources within those patches) and translocation on these disease dynamics, we ran simulations over the spatially separated network of topology B. Figure 7a shows the results of simulation runs where no attribute heterogeneity exists (all patch values are set equal). From this, we see that the separation into distinct spatial regions results in much greater initial bacteria growth, due to the limitations on the number of immune cells that can encounter the bacteria during the early stages on infection. An adaptive immune response begins to quell the infection, but in doing so it results in transfer of the bacteria to other areas of the lung. An oscillating process of bacterial growth occurs, with bacteria numbers increasing and decreasing over time, but never reaching the levels of the initial growth explosion. This can be seen as a latent form of TB: the bacteria levels are contained but not eradicated. As all patches contain the same values for their attributes, the bacteria levels at each patch remain approximately the same. The bacterial levels here far outweigh those seen in the single-patch model, except in the case where perfusion is drastically reduced.

**Fig. 8:**
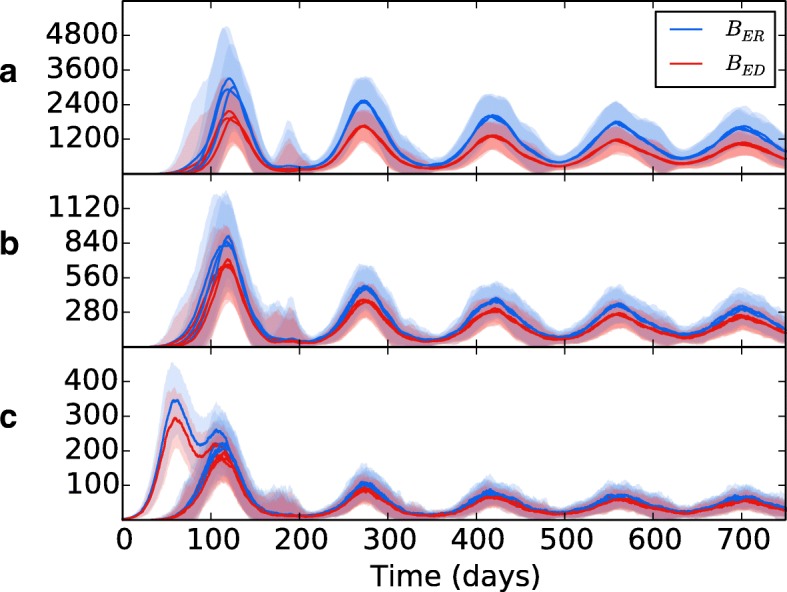
The bacterial levels of *B*_*ER*_ and *B*_*ED*_ in **a** apical patches, **b** middle patches and **c** basal patches. Line is mean, shading is standard deviation. All three regions show similar levels between replicating (blue) and dormant (red) bacteria, with a slight majority to replicating bacteria in all

Wethen introduced spatial heterogeneity to the different zones, with values as per Table 2, the results of which are shown in Fig. 7b. As per the previous experiment, bacterial growth occurs in the initial patch until the introduction of an adaptive immune response, which brings the bacterial levels under control but at the consequence of spreading bacteria to other regions. However, in this scenario the bacteria have access to the oxygen-rich, immune-poor regions at the lung apices, and once there the population explodes dramatically, reaching levels far higher than it did at the basal regions during initial infection. Again, the adaptive immune response is able to bring the bacterial levels down and reach an oscillating equilibrium. But in this scenario, we show that the levels of bacteria at latency are different for each region, with far higher levels being established at the apical regions than at the basal and middle regions. Figure 8 shows the levels of replicating and dormant bacteria in each region of the multi-patch network. We see that there are similar levels of replicating and dormant bacteria, with a slight majority to replicating bacteria in all patches.

### Bacterial translocation

To investigate the effects of translocation within the lung on bacterial growth, we ran experiments using different weight values on the LungEdges connecting the patches within topology B. Figure 7b shows the effects of no translocation within the lung (all WEIGHT values set to 0), whilst Fig. 7c shows the effects with translocation allowed between patches which reside in the same lobe (WEIGHT attribute of edges between patches in the same lobe is set to 1.0, and 0.0 for all other edges). Figure 7d shows the effects of allowing translocation between all areas of the lung. WEIGHT values are set as per Table 7.

**Table 7:** WEIGHT values for edges between patches in different lobes

	superior	Middle	Inferior
Superior	1.0	0.5	0.25
Middle	-	1.0	0.5
Inferior	-	-	1.0

**Fig. 9:**
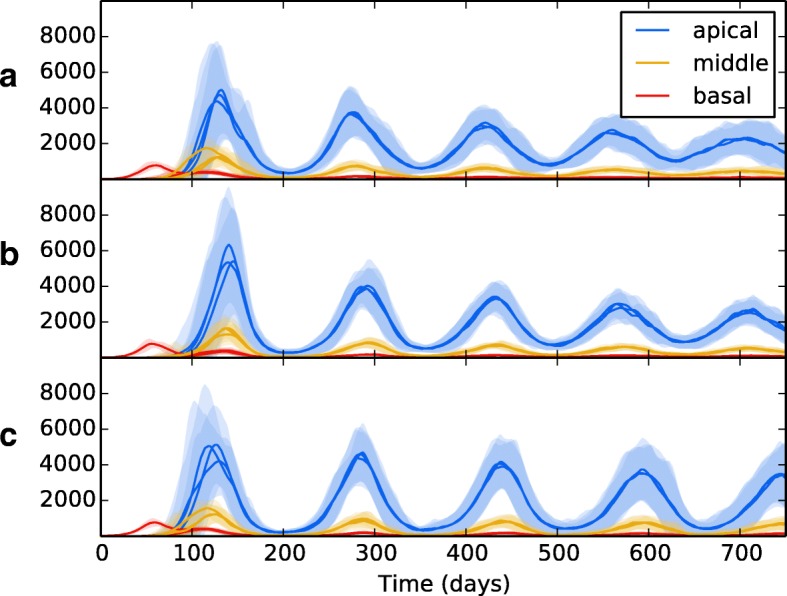
The overall bacterial growth (*B*_*ER*_, *B*_*ED*_, *B*_*IM*_ and *B*_*ID*_) upon a multiple patch topology with different routes of bacterial dissemination. Patches are grouped by spatial location (base, middle and apex). Line is mean, shading is standard deviation. All simulations use event parameters as per Tables 3, 4, 5 and 6. **a** shows the results of a network where translocation within the lung can only occur within the same lobe (i.e. small-distance translocation). **b** shows the effects of completely removing translocation within the lung. **c** shows the effects of removing translocation of bacteria via the lymphatics and bloodstream back to the lung. In all cases, removing options of translocation results in a similar pattern of growth to when all methods are viable (Fig. 7)

In Fig. 9, we tested the effects of removing a) long-scale inter-lung transmission b) all inter-lung transmission and c) bloodstream transmission from the lymphatics to the lung. In all cases, the overall pattern of bacterial growth in the lungs remains fairly robust, with minor changes in the amplitude of the resulting oscillations of bacteria levels. These findings suggest that the means of translocation across the pulmonary system are not as important as the heterogeneity therein. So long as bacteria can reach the favourable apical locations in some manner, they will be able to proliferate and establish a greater infection there.

## Discussion

Our results have shown that differentials in the lung environment, such as perfusion and oxygen tension, can drastically alter the levels of bacteria present once the immune system has contained the initial infection and established a latent form of disease. The heterogeneity of the lung environment creates a region at the apices that is highly oxygenated, thus affording the bacteria the opportunity to become highly replicating, and poorly perfused, thus providing less resistance to bacterial growth by immune cells; and we have shown that these factors significantly contribute to the progression of disease within our simulations. This demonstrates the necessity of *in silico* modelling of TB infections to take a broad view of the disease as it occurs within the lung: environmental factors play an important role in determine the scale of infection before latency is established. Developing new treatment regimens requires a better understanding of exactly which environments bacteria persist in within the lungs - computational models such as the one introduced here can help shed light on these niches.

The differences in routes of bacterial translocation across the pulmonary system appear to have little effect on the disease outcome; so long as bacteria have some means of reaching the preferential apical regions (i.e. through dissemination in the blood after establishing lymphatic disease or via direct transfer within the air of the lung), this will be enough to cause an enhanced population growth of bacteria in the region due to its favourable environment.

Figure 8 indicates that the bacterial population in all regions is composed of both replicating and dormant bacteria in almost equal measure. Whilst it is believed that a latent form of infection involves the host maintaining a population of dormant, non-replicating bacteria (Nuermberger et al. 2004), there is evidence that some bacterial replication occurs during latency (Karakousis 2014; Houk et al. 1968), which may explain the presence of a population of *B*_*ER*_ bacteria after initial infection and the oscillating bacteria levels in our model. However, the current model uses the environmental oxygen levels as the only influence on the switch between replication and dormancy - in reality, a wide range of factors are believed to initiate dormancy, including oxygen deficiency, nutrient deficiency, hyper-acidity and antibiotics (Lipworth et al. 2016). These stresses may drive bacteria to dormancy all over the lung, with the oxygen availability at the apices then providing the ideal environment for future reactivation. Future models should incorporate more of these factors to better mimic the dormancy phenomenon of Mtb.

This model is a highly abstracted representation of the actual conditions found during a TB infection, but it is nevertheless based on gross-scale real world pathology. These abstractions are necessitated by the current lack of understanding of how the disease pathology is influenced by its surroundings. We have shown that the introduction of a small amount of heterogeneity, by dividing the lung into a network consisting of 10 nodes, can have significant impact on the results of simulations in contrast with models that treat the lung as a homogeneous environment.

The purpose of this model is not, presently, to be predictive; it serves to provide insight into the complex factors that drive TB infections to their apical localisation. This insight can provide the basis for models which can build on this work to further study the impact of lung environment on TB. We have identified some parameters whose spatial heterogeneity is significant to apical localisation (such as reduced perfusion at the apices), but others may exist. Where possible, we have used parameters taken directly from the scientific literature.

## Conclusion

TB mortality represents a heavy burden on global health, with over 1 million people dying from the disease each year. In order to reduce this mortality, improved treatment regimens are desperately needed, but creating novel treatments requires a greater understanding of the environments within the lung in which bacteria proliferate and disease is established. Previous trials of new regimens have been unsuccessful possibly due to differential distribution of drugs into TB lesions (Prideaux et al. 2015). Understanding how the heterogeneous environment within the lungs contributes to disease formation is an important first step in creating treatment regimens with greater efficacy over shorter timeframes, thus improving patient adherence and reducing mortality and reducing the emergence of of drug-resistant strains of bacteria.

*In silico* modelling of TB disease has been used previously to model a single lesion of TB (Segovia-Juarez et al. 2004; Bowness et al. 2018) and to look at bacterial proliferation over the whole lung and the lymphatic system (Marino and Kirschner 2004; Marino et al. 2010). In this work, we have built what we believe to be the first *in silico* in-host model of tuberculosis that incorporates environmental heterogeneities to determine the impact they have of disease formation. To do this, we have chosen to use a networked metapopulation model whereby the alveolar tissue in the lung (where interactions between the bacteria and immune system occur) is modelled as an interconnected series of patches, each of which are given attribute values for environmental factors such as oxygen and perfusion based on their vertical position in the lung, thus creating a heterogeneous environment. We have shown that even a simple model with modest heterogeneity of environment has profound effects on the bacterial loads present in the body: the increased oxygen tension and reduced perfusion at the apical regions of the lung provides bacteria with an ideal niche in which to switch to a replicating cell-state, in a location where a strong immune response is difficult to establish. These findings can provide the groundwork for future modelling efforts which can incorporate more sophisticated dynamics and heterogeneities to investigate the impact the environment within the human lung has on both disease and its subsequent treatment.

Future iterations of the model will expand upon the work presented here; in particular, we hope to establish which specific processes during an infection drive bacterial dissemination to apices of the lungs and thus give rise to the apical localisation of post-primary TB, as well as which processes result in the oscillating bacterial levels seen. As the dynamics during infection are currently poorly understood, providing insight requires understanding which model parameters are significant in creating the dynamics seen in our model results. To do this, model reduction and sensitivity analysis will be required (Marino et al. 2008). Understanding which parameters are crucial to the emergent dynamics of the system can serve as a spur to further laboratory work, clinical trials or *in silico* work to refine these parameters.

Furthermore, more sophisticated anatomical dynamics can be included, through the use of more complex networks and space-filling trees to mimic the human bronchial tree structure and include a wider spectrum of environmental heterogeneities. Increasing the path length between patches may result in more heterogeneous patterns of bacteria spread across the network and thus translocation of bacteria may have a larger influence of disease outcome. Anatomical data should also be incorporated in order to provide a more accurate basis for the choice of attribute values.

We also aim to incorporate further aspects of a TB infection, such as a weakening of the immune system that occurs through immune-system suppressing drugs, human immunodeficiency virus (HIV) neoplasm or advancing age resulting in the deterioration of latent TB into post-primary TB. Additionally, the model will enable us to alter the pharmacokinetics and pharmacodynamics of multiple-drug chemotherapeutic treatments, and thus, investigate what effects lung heterogeneities have on the treatment of an established disease. If this development were to be successful, it would be a significant step towards a model that could be used to build a virtual clinical trial simulation.

## Additional file

Additional file 1 Supplementary Material. (PDF 97 kb)

## Abbreviations

AM: Alveolar macrophage
APC: Antigen-presenting cell
DC: Dendritic cell
HIV: Human immunodeficiency virus
Mtb: Mycobacterium tuberculosis
TB: Tuberculosis

## Footnotes

1 The Gillespie Algorithm is named after its creator, Daniel Gillespie, who is no relation to the author of this work.
