# Supplementary material for: A spatially heterogeneous network-based metapopulation software model applied to the simulation of a pulmonary tuberculosis infection
Source: Appl Netw Sci. 2018 Aug 23;3(1):33. doi: 10.1007/s41109-018-0091-2 (PMC6214320; doi:10.1007/s41109-018-0091-2)
Supplement: Supplementary file 1 — Supplementary Material. (PDF 97 kb) [file 41109_2018_91_MOESM1_ESM.pdf]

A spatially heterogeneous network-based metapopulation software model applied to the simulation of a pulmonary tuberculosis infection - Supplementary Material

MetapopPy

Network

The *Network* package reflects the environment which is being simulated; this takes the form of a network of nodes and edges. The nodes of the network are *Patches* and each of these contain a subsection of the total system population. These sub-populations are divided into distinct compartments - each of which has a count reflecting how many members of that compartment exist within the patch at the current time. A member may only belong to one compartment at any given time, but may switch to a different compartment dependent on the system dynamics and the compartment paradigms chosen (i.e two compartments may model a real-world species in different states, such as infected or susceptible in epidemic dynamics). Each patch may also contain a set of attributes, which allow for the creation of heterogeneous environments, with different areas of the environment having different resources which influence the interactions and translocations that occur upon or between them. The patches are joined by instances of an *Edge* class. Each edge indicates some form of possible dispersal of members from one patch to another. Edges may also include attributes, indicating some difference in the environment that favours or hinders movement in a specific direction. Edges may be set as bi-directional or directed, implying free movement between two patches or movement in only one direction, respectively.

Events

The *Events* package is used to simulate the interactions between individuals within the patches and translocation of members between patches. Event modelling within the MetapopPy system uses the Gillespie Algorithm[1] to perform

[1] The Gillespie Algorithm is named after its creator, Daniel Gillespie, who is no relation to the author of this work.

events asynchronously [1].

An event class is initialised with a reaction parameter (which can be interpreted as the rate of a single event occurrence in the specified unit time), and a state variable (which can be interpreted as the number of possible reactions in the network for the given event. The rate for the event is thus computed as the product of the reaction parameter and the state variable. When a change occurs on the network, each event must recalculate its rate by recalculating the state variable using the new population values.

Each event class must define a function to calculate its state variable by interrogating the network: this function determines the number of possible reactions for the given event type based on the contents of the patches and edges of the network. Each event instance is initialised with a reaction parameter (which can be interpreted as the rate of a single event occurrence in the specified unit time) and the event calculates its rate by multiplying the reaction parameter and the state variable.

Each event class must also define a *perform* function, which determines what changes are made to the subpopulations of the network when the event occurs. These functions are used within the Dynamics package. An event is classed at occurring at only one patch, but may update multiple patches. For example, movement from patch A to patch B is classed as occurring at patch A and depends only on the values of the population there (and the edges attached to patch A), but when performed, the event updates the population of both patches.

Dynamics

The *Dynamics* package joins a network with a series of events that enact upon it. When a simulation of the model is run, the network is seeded with initial conditions: a set of subpopulation compartment counts (determined by the user) and attributes for all patches. The simulation time is set initially at zero. MetapopPy then uses an asynchronous time modelling system following the Gillespie algorithm: each event calculates its rate as specified above and an event is chosen probabilistically based upon these rates, with a greater rate indicating a greater likelihood of being chosen. A timestep, τ, for this event to occur is then chosen using Equation 1 (as per [1]), with a being the total sum of the rate of all events, and r being a randomly generated number in the range (0,1).

$$\tau = \frac{1}{a} ln \left(\frac{1}{r} \right) \tag{1}$$

The chosen event is then performed, and updates the network in its defined manner. The simulation time is then incremented by τ and the process repeats, until a set time-limit is exceeded or there is no possibility of any event occurring (i.e. a=0). The sub-population values of all patches are recorded at a user-defined time interval throughout. When a patch is updated by an event, other events need to recalculate their state variables and their rates. To recalculate

the state variable contribution of all patches to all events would be inefficient, as some values may not have changed. Therefore, an *UpdateHandler* class is included to propagate updates efficiently. This class tracks which events occur at which patch, and also which compartments are used to calculate the state variable contribution for each event. When the compartments of a patch are updated, the update handler is triggered and only events which a) occur at the patch, and b) are dependent on the amended compartments, recalculate their state variable contribution from the amended patch. By tracking these dependencies and utilising them in this manner, the computation needed after each patch update is drastically reduced.

TBMetapopPy

Events

TBMetapopPy contains a number of events to simulate the growth of bacteria and the interactions between the immune cells of the body and the bacteria. These events are described in this section, and listed in tables 1, 2, 3 and 4, along with their respective reaction parameters, state variable equations and outcomes.

Bacteria events

An initial population of bacteria is deposited in the lung. These bacteria may change state depending on the environmental attributes of the patch, with lower values of O_2 at the patch increasing the probability of switching from B_{ER} to B_{ED} (event 4), and greater O_2 values increasing the probability of switching from B_{ED} to B_{ER} (event 5).

A bacterium may replicate producing a new member of the same compartment: replication of B_{ER} and B_{ED} is unrestricted (events 1 and 2 repsectively), whilst replication of B_{IM} (event 3) is reduced when the average population inside a cell approaches the carrying capacity of the cell. We assume that B_{ID} do not replicate due to the small capacity of the dendritic cell.

Extracellular bacteria may translocate: bacteria present in a LungPatch may translocate along the air (via LungEdge instances, events 6 and 7), with greater probability along edges with greater WEIGHT values. Extracellular bacteria in LymphPatches may translocate along a BloodEdge back into the lung (event 8), with the edge to move along chosen probabilistically based on the perfusion value.

Macrophage events

M_R members are recruited into the LungPatch (event 20) and LymphPatch (event 23) instances at a constant rate (with LungPatch recruitment scaled by the perfusion (Q) value of the patch) and die at a constant rate (event 31), resulting in an equilibrium population value in the absence of infection. When

bacteria are present, contact between macrophages and extracellular bacteria may result in a) the bacterium being destroyed (events 9 and 10) or b) the macrophage becoming infected (events 26 and 27), in which case an M_R member turns to M_I and the bacterium member changes to B_{IM}.

Rather than modelling cytokine dynamics directly, we simplify our model by using cytokine producing populations directly (i.e. M_I, M_A and TC_A) as the triggers for events which are dependent on these chemicals. Enhanced macrophage recruitment events (events 21, 22, 24 and 25) are examples of these, and in this model these events are triggered by the presence of M_I and M_A members, which cause more M_R members to be recruited to a patch. T-cell activation by antigen presentation may be done by any macrophage which has come into contact with bacteria and then absorbed antigens; in TBMetapopPy, we use only M_I cells as these cells (event 40), which may translocate from the LungPatches to the LymphPatches along LymphEdges (also transferring their load of B_{IM}, calculated as the average number of B_{IM} per M_I, shown in event 28).

M_I members may become overwhelmed by their internal population of B_{IM} bacteria, causing them to 'burst' (event 34), destroying the M_I member and releasing the average internal number of bacteria back out. We assume that the internal conditions of the macrophage are a stressful environment, and therefore all bacteria are released into the B_{ED} compartment, having become dormant to survive inside the immune cell.

Dendritic cell events

Dendritic cells act similarly to macrophages, and are recruited (event 18) and die (event 16) at constant rates, with recruitment of DC_I members being increased by the presence of extracellular bacteria (event 19).

Contact between DC_I and extracellular bacteria may cause uptake of a bacterium (events 13 and 14), converting DC_I to DC_M and the bacterium to B_{ID} (we assume dendritic cells do not destroy bacteria). DC_M members may translocate (event 15) to the LymphPatches along LymphEdges for antigen presentation.

T-cell events

A population of T_N members exists in the LymphPatch instances at the start of simulation. Like other immune cells, these cells die (event 42) and are replaced by newly recruited cells (event 36). As the infection spreads and APCs (M_I and DC_M) transfer to the lymphatics, these cells may trigger the naïve population to activate (events 39 and 40), converting T_N into T_A. These T_A members may then migrate back to the lung (event 41) along a BloodEdge, with the choice of edge linked to the perfusion value, resulting in easier travel to areas with greater perfusion.

Activated T-cells have two main functions in the model: the first is to trigger activation of macrophages (converting M_R to M_A, event 29). Activated

macrophages will naturally revert to a resting state (event 30) if the t-cell presence is too low.

Secondly, T-cells cause the cytotoxic destruction of infected macrophages (event 35). The M_I macrophage is destroyed, and likewise any B_{IM} bacteria inside are also destroyed. These functions may be performed at both classes of patch.

References

[1] Gillespie, D.T.: A general method for numerically simulating the stochastic time evolution of coupled chemical reactions. Journal of Computational Physics **22**(4), 403–434 (1976). doi:10.1016/0021-9991(76)90041-3

Table 1: Bacterial events within *TBMetapopPy*. Param: reaction parameter, State variable: the function used to calculate the state variable contribution of each patch to the event, Outcome: the changes made to the patch when the event is chosen to be performed

	**Event name**	**Description**	**Param**	**State variable**	**Outcome**
1	Bacterial replication (B_{ER})	A B_{ER} bacterium replicates, creating an identical bacterium	R_{ER}	B_{ER}	B_{ER}: 1
2	Bacterial replication (B_{ED})	A B_{ED} bacterium replicates, creating an identical bacterium	R_{ED}	B_{ED}	B_{ED}: 1
3	Bacterial replication (B_{IM})	A B_{IM} replicates within a macrophage, creating an identical bacterium	R_{IM}	$B_{IM} * \left(1 - \frac{{B_{IM}}^{\theta}}{{B_{IM}}^{\theta} + (M_{Cap} * M_I)^{\theta}}\right)$	B_{IM}: 1
4	Bacterial state change (B_{ER} to B_{ED})	A replicating bacterium becomes dormant due to lack of oxygen	C	$B_{ER} * \frac{{O_2}^{-\lambda}}{{H_C}^{-\lambda} + {O_2}^{-\lambda}}$	B_{ER}: -1, B_{ED}: 1
5	Bacterial state change (B_{ED} to B_{ER})	A dormant bacterium reactivates due to the availability of oxygen	C	$B_{ED} * \frac{{O_2}^{\lambda}}{{H_C}^{\lambda} + {O_2}^{\lambda}}$	B_{ED}: -1, B_{ER}: 1
6	Bacterial translocation (lung - B_{ER})	A replicating bacterium moves along the air within the lung	T_{B_Lung}	B_{ER}	Patch 1: B_{ER}: -1 Patch 2: B_{ER}: 1
7	Bacterial translocation (lung - B_{ED})	A dormant bacterium moves along the air within the lung	T_{B_Lung}	B_{ED}	Patch 1: B_{ED}: -1 Patch 2: B_{ED}: 1
8	Bacterial translocation (blood - B_{ED})	A dormant bacterium moves along the bloodstream from the lymphatics to the lung	T_{B_Blood}	B_{ED}	LymphPatch: B_{ED}: -1 LungPatch: B_{ED}: 1
9	Bacterial destruction (B_{ER} by M_R)	An M_R macrophage destroys a B_{ER} bacterium	D_{B_R}	$B_{ER} * M_R$	B_{ER}: -1
10	Bacterial destruction (B_{ED} by M_R)	An M_R macrophage destroys a B_{ED} bacterium	D_{B_R}	$B_{ED} * M_R$	B_{ED}: -1
11	Bacterial destruction (B_{ER} by M_A)	An M_A macrophage destroys a B_{ER} bacterium	D_{B_A}	$B_{ER} * M_A$	B_{ER}: -1
12	Bacterial destruction (B_{ED} by M_A)	An M_A macrophage destroys a B_{ED} bacterium	D_{B_A}	$B_{ED} * M_A$	B_{ED}: -1

Table 2: Dendritic cell events within *TBMetapopPy*. Param: reaction parameter, State variable: the function used to calculate the state variable contribution of each patch to the event, Outcome: the changes made to the patch when the event is chosen to be performed

	**Event name**	**Description**	**Param**	**State variable**	**Outcome**
13	Dendritic cell maturation by B_{ER}	An immature dendritic cell comes into contact with a B_{ER} bacterium, uptakes it and matures	U	$DC_I * B_{ER}$	DC_I: -1, DC_M: 1
14	Dendritic cell maturation by B_{ED}	An immature dendritic cell comes into contact with a B_{ED} bacterium, uptakes it and matures	U	$DC_I * B_{ED}$	DC_I: -1, DC_M: 1
15	Dendritic cell translocation	A mature dendritic cell migrates from the lung to the lymphatics	T_D	$DC_M * DRAINAGE$	LungPatch: DC_M: -1 LymphPatch: DC_M: 1
16	Dendritic cell death (DC_I)	An immature dendritic cell dies	D_{DCI}	DC_I	DC_I: -1
17	Dendritic cell death (DC_M)	A mature dendritic cell dies	D_{DCM}	DC_M	DC_M: -1
18	Dendritic cell recruitment	An immature dendritic cell is recruited into the lung	R_D	Q	DC_I: 1
19	Dendritic cell recruitment (enhanced)	Recruitment of immature dendritic cells into the lung is increased by cytokines given off by bacteria	E_D	$Q * \left(\frac{B_{ER}+B_{ED}}{B_{ER}+B_{ED}+H_D}\right)$	DC_I: 1

Table 3: Macrophage events within *TBMetapopPy*. Param: reaction parameter, State variable: the function used to calculate the state variable contribution of each patch to the event, Outcome: the changes made to the patch when the event is chosen to be performed

	**Event name**	**Description**	**Param**	**State variable**	**Outcome**
20	Macrophage recruitment (lung, standard)	A macrophage is recruited into the lung	R_{M_lung}	Q	M_R: 1
21	Macrophage recruitment (lung, enhanced by M_A)	Macrophage recruitment into the lung is enhanced by cytokines given off by M_A	E_{M_MA}	$Q * M_A$	M_R: 1
22	Macrophage recruitment (lung, enhanced by M_I)	Macrophage recruitment into the lung is enhanced by cytokines given off by M_I	E_{M_MI}	$Q * M_I$	M_R: 1
23	Macrophage recruitment (lymph, standard)	A macrophage is recruited into the lymphatics	R_{M_lymph}	1	M_R: 1
24	Macrophage recruitment (lymph, enhanced by M_A)	Macrophage recruitment into the lymphatics is enhanced by cytokines given off by M_A	E_{M_MA}	M_A	M_R: 1
25	Macrophage recruitment (lymph, enhanced by M_I)	Macrophage recruitment into the lymphatics is enhanced by cytokines given off by M_I	E_{M_MI}	M_I	M_R: 1
26	Macrophage bacterial uptake (B_{ER})	An M_R macrophage takes up a replicating bacteria, and becomes infected	I	$M_R * \left(\frac{B_{ER}}{B_{ER}+B_{ED}+H_{MI}}\right)$	M_R: -1, B_ER: -1, M_I: 1, B_{IM}: 1
27	Macrophage bacterial uptake (B_{ED})	An M_R macrophage takes up a dormant bacteria, and becomes infected	I	$M_R * \left(\frac{B_{ED}}{B_{ER}+B_{ED}+H_{MI}}\right)$	M_R: -1, B_ED: -1, M_I: 1, B_{IM}: 1
28	Macrophage translocation	An M_I macrophage migrates from the lung to the lymphatics	T_M	$M_I * DRAINAGE$	LungPatch: M_I: -1 LymphPatch: M_I: 1
29	Macrophage activation	An M_R macrophage enters an activated state triggered by T_A	A_M	$M_R * \left(\frac{T_A}{T_A+H_{MA}}\right)$	M_R: -1, M_A: 1
30	Macrophage deactivation	An M_A macrophage returns to a deactivated state	A_M	$M_R * \left(\frac{H_{MA}}{T_A+H_{MA}}\right)$	M_R: -1, M_A: 1
31	Macrophage death (M_R)	An M_R macrophage dies	D_{MR}	M_R	M_R: -1
32	Macrophage death (M_A)	An M_A macrophage dies	D_{MA}	M_A	M_A: -1
33	Macrophage death (M_I)	An M_I macrophage dies	D_{MI}	M_I	M_I: 1
34	Macrophage bursting	An M_I macrophage is overwhelmed by its internal bacterial load and bursts open, destorying the macrophage	B	$M_I * \left(\frac{{B_{IM}}^{\epsilon}}{{B_{IM}}^{\epsilon}+(M_I*M_{Cap})^{\epsilon}}\right)$	M_I: -1, B_{IM}: -1 * $\frac{B_{IM}}{M_I}$, B_{ED}: $\frac{B_{IM}}{M_I}$

Table 4: T-cell events within *TBMetapopPy*. Param: reaction parameter, State variable: the function used to calculate the state variable contribution of each patch to the event, Outcome: the changes made to the patch when the event is chosen to be performed

	**Event name**	**Description**	**Param**	**State variable**	**Outcome**
35	Macrophage destroyed by T-cell	An infected macrophage is destroyed by a T-cell	K	$M_I * \left(\frac{\frac{T_A}{M_I}}{\frac{T_A}{M_I} + H_K} \right)$	M_I: -1, B_{IM}: -1 * $\frac{B_{IM}}{M_I}$
36	T-cell recruitment (standard)	A naive T-cell is recruited into the lymphatics	R_T	1	T_N: 1
37	T-cell recruitment (enhanced by DC_M)	Recruitment of naive T-cells into the lymphatics is enhanced by cytokines given off by DC_M	E_{T_DCM}	DC_M	T_N: 1
38	T-cell recruitment (enhanced by M_I)	Recruitment of naive T-cells into the lymphatics is enhanced by cytokines given off by M_I	E_{T_MI}	M_I	T_N: 1
39	T-cell activation by DC_M	DC_M cells present antigens to naive T-cells, causing them to become activated	A_{T_DCM}	$T_N * DC_M$	T_N: -1, T_A: 1
40	T-cell activation by M_I	M_I cells present antigens to naive T-cells, causing them to become activated	A_{T_MI}	$T_N * M_I$	T_N: -1, T_A: 1
41	T-cell translocation	An activated T-cell migrates from the lymphatics, through the bloodstream into the lung	T_T	T_A	LymphPatch: T_A: -1 LungPatch: T_A: 1
42	T-cell death (T_N)	A naive T-cell dies	D_{TN}	T_N	T_N: -1
43	T-cell death (T_A)	An activated T-cell dies	D_{TA}	T_A	T_A: -1
